# Use of Services by People Living Alone With Cognitive Impairment: A Systematic Review

**DOI:** 10.1093/geroni/igab004

**Published:** 2021-01-18

**Authors:** Amy Rosenwohl-Mack, Leslie Dubbin, Anna Chodos, Sarah Dulaney, Min-Lin Fang, Jennifer Merrilees, Elena Portacolone

**Affiliations:** 1 Department of Social and Behavioral Sciences, School of Nursing, University of California San Francisco, USA; 2 Division of Geriatrics, University of California San Francisco, USA; 3 Division of General Internal Medicine, Zuckerberg San Francisco General Hospital, University of California San Francisco, USA; 4 Memory and Aging Center, University of California San Francisco, USA; 5 Library, University of California San Francisco, USA; 6 Institute for Health & Aging, University of California San Francisco, USA

**Keywords:** Dementia, Health care services, Home care services, Living arrangements, Long-term care

## Abstract

**Background and Objectives:**

Formal supports and social services are essential to people living alone with cognitive impairment (PLACI) because they are at risk of negative health outcomes and lack cohabitants who may support them with cognitively demanding tasks. To further our understanding of this critical and worldwide issue, we conducted a systematic review to understand whether, and how, PLACI access and use essential formal supports and services.

**Research Design and Methods:**

We searched 6 databases (PubMed, Embase, PsycINFO, CINAHL, Web of Science, and Sociological Abstracts) to identify quantitative and mixed-method literature on formal service use among PLACI. The initial search was conducted in 2018 and updated in 2020.

**Results:**

We identified 32 studies published between 1992 and 2019, representing 13 countries, that met our criteria: 16 reported on health services and 26 on social services. Most studies compared PLACI with people with cognitive impairment living with others. Health service use was lower or similar among PLACI, as opposed to counterparts living with others. Most studies reported a higher use of social services (e.g., home services) among PLACI than those living with others. Overall use of essential home service among PLACI was higher in Europe than in the United States, a country where large portions of PLACI were reported receiving no formal services.

**Discussion and Implications:**

We identified wide variability among countries and major gaps in service use. Results for use of health services were mixed, although our findings suggest that PLACI may have fewer physician visits than counterparts living with others. Our findings suggest that varying policies and budgets for these services among countries may have affected our findings. We encourage researchers to evaluate and compare the influence of social policies in the well-being of PLACI. We also encourage policy makers to prioritize the needs of PLACI in national dementia strategies.

**Translational Significance:** This article highlights gaps in the provision of essential services and supports to people living alone with cognitive impairment. International comparisons point to the importance of establishing social policies to ensure access to essential formal services and supports for this at-risk population with unique needs. This article also underscores the need for further investigations into access to and use of essential formal services and support. This article illuminates the need for social policies promoting the identification, proactive outreach, and tailored, coordinated, and state-subsidized support of all people with cognitive impairment living alone worldwide.

## Background

Living alone in old age is common worldwide. In Europe and North America, one third of older adults (60 and older) live alone (United Nations, 2017). The likelihood of this living arrangement increases with age. For example, in Sweden almost half of adults aged 75 and older live alone (Shaw et al., 2018; additional statistics in the Supplementary Material). Many older adults living alone are likely to have cognitive impairment, which is a condition that also increases with age. Cognitive impairment is an umbrella term that includes mild cognitive impairment and Alzheimer’s disease and related dementias. International estimates of people living alone with cognitive impairment (PLACI), the vast majority of whom are older adults, range between 28% and 55% (Clare et al., 2020; Edwards et al., 2020; Eichler et al., 2016; Michalowsky et al., 2016b). These estimates are tentative because PLACI are often undiagnosed or diagnosed late (Gould et al., 2010; Lehmann et al., 2010).

For people with cognitive impairment, living alone represents an opportunity to remain in a familiar home environment where they are less disoriented (Duane et al., 2013). However, this living arrangement is also associated with greater health risks and unmet needs compared to living with others. Specifically, previous studies have shown that PLACI are at high risk for health threats including self-neglect (Charles et al., 2015; Miranda-Castillo et al., 2010), untreated medical conditions (Cermakova et al., 2017; Meaney et al., 2005), and extreme isolation (Portacolone et al., 2021) compared to those living with cohabitants (e.g., spouses, adult children).

Access to formal supports and services is essential to PLACI (Fazio et al., 2018) for two reasons. First, PLACI are likely to lack informal caregivers because they usually reside in western countries that have low informal caregiver ratios. For example, the ratio of people aged 65 and older versus people aged 15–64 is estimated at 3.4 in Europe and 3.9 in North America, as opposed to 16 in Africa and 7.6 in Asia (United Nations, 2019). Second, independent of their country of residence, PLACI lack cohabitants who might help with crucial but cognitively demanding skills, such as managing appointments and medications, paying bills, and buying groceries. PLACI are particularly at risk of not receiving care because informal and unpaid care is often the most common type of care worldwide. For example, the costs of unpaid care provided by family members and others in the community make up 40%–70% of the total costs of dementia worldwide (OECD, 2018; World Dementia Council, 2013). Overall, formal supports and services that are essential to PLACI include acute medical care, as well as long-term care (i.e., home care aides, adults day programs, meal delivery) because cognitive impairment usually lasts for decades. As their cognitive impairment increases, people living alone are likely to require more assistance. For example, those with mild cognitive impairment may need support with strategies to help them maintain independence (e.g., calendar, task list, pill box, automatic bill pay). Those with more advanced cognitive impairment may benefit from more instrumental help (e.g., assistance with laundry, meal preparation, medications management).

Social policies influence access to these essential services because they include regulations on whether and how these services are publicly available. Countries with robust welfare policies and universal health care usually provide these services publicly. For example, in Denmark, older Danes living alone can access state-subsidized home care aides rigorously trained in dementia care care—a service essential to them—soon after they receive a diagnosis of cognitive impairment (Portacolone, 2018). State-subsidized home aides are also available to PLACI in Germany, Japan, Canada, France, and Scandinavia, among other countries (Campbell et al., 2010). Access to public home care aides is more limited in countries that rely heavily on the private sector for their long-term care. In the United States, for example, public home care aides are only available to a fraction of PLACI whose income is low enough to qualify for Medicaid, the national public insurance for low-income U.S. residents. As a result, most PLACI need to privately pay for essential formal long-term supports, which is often unaffordable to them over time (Shih et al., 2014).

It is critical to learn whether, and how, PLACI access and use formal supports and services for three reasons. First, these services are essential to them. Second, the presence of social policies granting access to these services does not automatically imply that these essential services are easily accessed or used over time. Finally, understanding service gaps is especially important during the coronavirus disease 2019 pandemic, as PLACI are at particular risk for adverse health outcomes during public health crises (Holmes et al., 2020; Koff & Williams, 2020; Portacolone et al., 2021).

Yet no previous systematic review focuses on PLACI, thereby limiting our understanding of their needs and the best ways to support their health and well-being. One systematic review evaluated the use of services by older adults with cognitive impairment in the United States; however, the authors did not report any findings on those living alone (Weber et al., 2011). Bieber et al.’s (2019) recent scoping review of factors influencing access to and the use of services in dementia pointed to the influence of national social policies to support PLACI. Several published systematic reviews have assessed health-promoting interventions for older adults living alone, without focusing on those with cognitive impairment (Haslbeck et al., 2012; Ilgaz & Gözüm, 2019; Song & van der Cammen, 2019; Tamminen et al., 2019). Given this gap in the literature and the high levels of unmet needs reported among PLACI, we conducted a systematic review to answer the following question: What is known about the use of formal health and social services among PLACI worldwide?

## Method

We followed PRISMA guidelines in conducting and reporting this systematic review (Moher et al., 2009); PROSPERO registration number CRD42019118315. While conducting initial exploratory searches, we noted that many studies that met our inclusion criteria did not include the word “service(s)” in their titles or abstracts, and referred instead to specific types of services. In order to maximize sensitivity and avoid missing important findings, we followed a two-stage process. First, we conducted a systematic literature search and screened the retrieved citations to identify all publications relating to PLACI. Next, we rescreened the resulting studies to identify those quantitative and mixed-methods studies that specifically investigated use of services as further detailed in the *Eligibility Criteria* section.

### Information Sources and Search Strategy

In collaboration with a medical librarian (M.-L. Fang), we conducted a comprehensive search of six electronic databases (PubMed, Embase, PsycINFO, CINAHL, Web of Science, and Sociological Abstracts) in August 2018 (see Supplementary Material). We drew on broad initial iterative searches and the CareSearch dementia PubMed filter (CareSearch, 2017) to inform the search strategy. No date limits were applied, in order to track the evolution of research in this field over time. We used Web of Science to find citing, cited, and relevant references from studies selected for inclusion, and we manually searched five key journals: *The Gerontologist*, *Alzheimer’s & Dementia*, *Dementia*, *American Journal of Alzheimer’s Disease and Other Dementias*, and the *International Journal of Geriatric Psychiatry*. Members of the review team also used internet searches and expert consultation to identify potential grey literature. Grey literature was included to broaden the scope of our review. Specifically, we wanted to ensure that we included a full range of evaluations, as well as studies with null findings that are often less likely to be published in peer-reviewed journals (Adams et al., 2016; Mahood et al., 2014). To ensure our findings would be current, in January 2020 we repeated the search from the same sources.

### Eligibility Criteria

In the rescreening process, we included all English-language quantitative and mixed-methods studies presenting primary data on PLACI. We excluded qualitative studies because we wanted to report on generalizable trends identified through quantitative analyses (Creswell & Plano Clark, 2018). The status of cognitive impairment was considered as a criterion because we wanted to examine access to services for PLACI. We accepted any diagnostic criteria for dementia or cognitive impairment (i.e., impairment that does not meet criteria for dementia), but excluded studies that only measured subjective cognitive decline or subclinical memory problems associated with typical aging. Studies with a primary focus on other conditions associated with cognitive decline such as traumatic brain injury, stroke, Parkinson’s disease, and schizophrenia were also excluded, due to their distinct patterns of functional and psychological complications.

Living alone was defined as living in a noninstitutional setting without others; we excluded assisted living facilities, nursing homes, and continuing care retirement communities. We included studies where PLACI were compared to those with cognitive impairment living with others, those living alone without cognitive impairment, as well as studies with no comparison group. We excluded from the review studies in which living alone and having cognitive impairment were two predictors in a multivariable model, but the subgroup of participants meeting both of these criteria was not analyzed separately. Eligible studies either focused specifically on PLACI or included an identifiable subgroup of such participants *and* reported quantitative findings on their use of services. Services were defined broadly, including, but not limited, to inpatient and outpatient health care, emergency services, home health care, assistance within the home, and community services. Only formal service use was considered. We excluded studies that only reported on unpaid caregiving because we focused on formal services. Finally, we accepted any type of reporting (e.g., self-report, proxy report, as well as medical records data).

### Study Selection and Data Management Process

**Figure 1.**
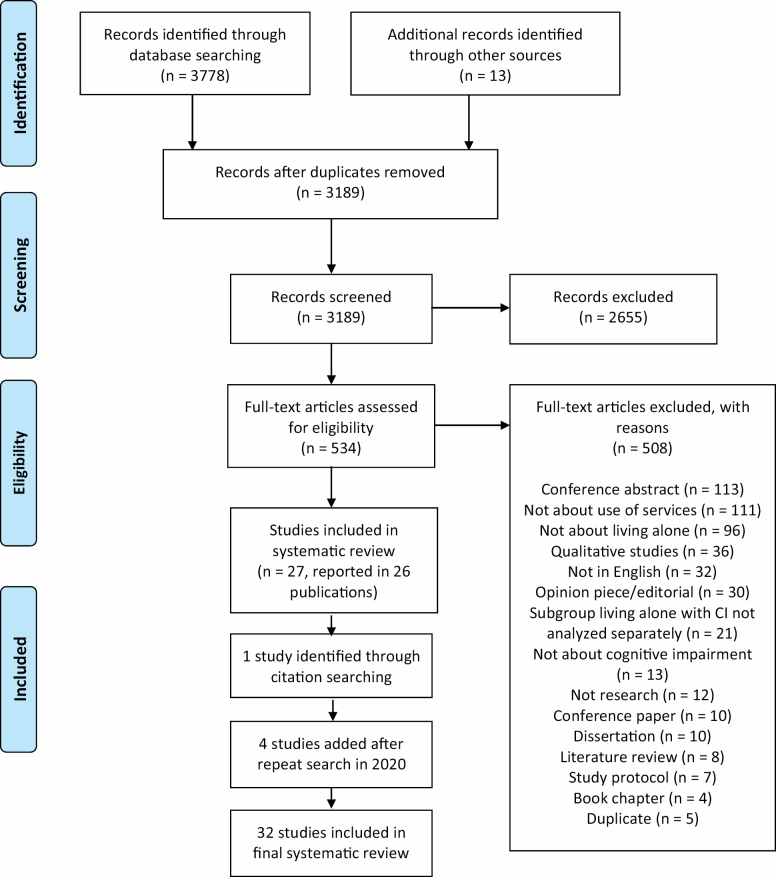
PRISMA diagram. From Moher et al. (2009).

A PRISMA flow diagram (see Figure 1) was used to illustrate the selection processes and results. All retrieved studies were imported into Endnote reference management software (Clarivate Analytics, Endnote X8.2, 2018) for deduplication, and the remaining citations were uploaded to Covidence (Veritas Health Innovation, Covidence Systematic Review Software, 2013). Two independent reviewers (A. Rosenwohl-Mack and L. Dubbin) screened study titles and abstracts against the inclusion and exclusion criteria, followed by full-text review of remaining studies. The two reviewers discussed any discrepancies, and in the case of unresolved conflicts a third reviewer (E. Portacolone) made the final decision, after group discussion.

### Data Items and Data Collection Process

One team member extracted study characteristics, including description of references (e.g., authors, publication date, aims), sociodemographics (e.g., location/setting, age, race/ethnicity, gender, diagnosis), study design, comparison group, and results. A second reviewer cross-checked the accuracy of the extracted data.

### Risk of Bias in Individual Studies

We used Joanna Briggs Institute (JBI) Checklists for Cohort Studies, Case Series, and Analytical Cross Sectional Studies to assess study quality and risk of bias (Aromataris & Munn, 2020). Two researchers independently assessed each study (A. Rosenwohl-Mack and L. Dubbin), and differences of opinion were resolved through discussion, with involvement of a third team member as necessary. Incorporating grey literature into systematic reviews can present challenges, because it is not subject to the strict reporting criteria imposed by academic journals (Adams et al., 2017). As a result, we applied JBI quality assessment criteria to all studies, and for grey literature we also made a qualitative assessment of the data sources and methods used. Specifically, first we assessed author credentials, data sources, and publication location. Next, we reached consensus on whether or not the grey literature was of sufficient quality to be included (Adams et al., 2017).

## Results

### Study Selection

From the search conducted in 2018, we identified 26 relevant publications, reporting on 27 unique studies (one paper reported on two separate studies). During the data extraction process, we identified one additional relevant study. In 2020, we added four studies after we repeated the search: two published in 2018 and two in 2019. In total, 32 studies were included (Figure 1). The research team identified 13 grey literature publications relating to PLACI; only one was eligible for inclusion and had already been identified as it was also published in a peer-reviewed journal.

### Quality Assessment Results

Overall, the included studies demonstrated robust research designs and thorough reporting (see Supplementary Material), except for an Alzheimer’s Association (2012) report. In this report we were unable to assess many of the JBI criteria because of the limited reporting of methods. However, the findings were based on Medicare data, the analysis was conducted by a health policy professor, and the report was prepared by PhD-trained epidemiologists. Thus, we included this report in the review but identified it as grey literature of uncertain quality in the findings.

### Study Characteristics

The 32 studies were published between 1992 and 2019, representing 95,424 participants. Detailed characteristics of the included studies are outlined in Table 1 in the Supplementary Material. Thirteen countries were represented: United Kingdom (*n* = 7; Chi et al., 1995; Gage et al., 2015; Henderson et al., 2019; Knapp et al., 2016; Miranda-Castillo et al., 2010; Schneider et al., 2002), United States (*n* = 5; Alzheimer’s Association, 2012; Bass et al., 1992; Edwards & Morris, 2007; Gibson & Richardson, 2017; Webber et al., 1994), Sweden (*n* = 5; Larsson et al., 2004, 2006; Måvall & Malmberg, 2007; Wattmo et al., 2011, 2014), Canada (*n* = 4; Ebly et al., 1999; Thiruchselvam et al., 2012; Tuokko et al., 1999), Germany (*n* = 3; Eichler et al., 2016; Michalowsky et al., 2016b, 2018), Japan (*n* = 2; Lin et al., 2017; Takechi et al., 2012), France (*n* = 2; Nourhashemi et al., 2005; Soto et al., 2015), Australia (*n* = 1; Rahja et al., 2018), Ireland (*n* = 1; O’Brien et al., 2019), and Norway (*n* = 1; Moholt et al., 2020). One European study included participants from the Netherlands, Germany, the United Kingdom, Ireland, Sweden, Norway, Italy, and Portugal (Kerpershoek et al., 2019). The mean age of the samples ranged from 74.2 to 86.9 years, and in all but two studies the majority of participants were female. Where demographics were reported separately, the living alone group typically had a higher proportion of female participants and a higher mean age. Race/ethnicity data were only reported in seven studies (Bass et al., 1992; Edwards & Morris, 2007; Gibson & Richardson, 2017; Knapp et al., 2016; Schneider et al., 2002, 2003; Webber et al., 1994); in one of these, the sample was 100% African American (Edwards & Morris, 2007), and the remaining six were majority White (Edwards & Morris, 2007; Knapp et al., 2016; Schneider et al., 2002, 2003; Webber et al., 1994). Out of the 32 studies, 18 included any dementia diagnosis (Alzheimer’s Association, 2012; Ebly et al., 1999; Edwards & Morris, 2007; Eichler et al., 2016; Gage et al., 2015; Henderson et al., 2019; Kerpershoek et al., 2019; Knapp et al., 2016; Michalowsky et al., 2018; Miranda-Castillo et al., 2010; Moholt et al., 2018; Nourhashemi et al., 2005; O’Brian et al., 2017; Rahja et al., 2018; Schneider et al., 2002, 2003; Soto et al., 2015; Webber et al.,1994), seven included people with any kind of cognitive impairment (Bass et al., 1992; Chi et al., 1995; Gibson & Richardson, 2017; Michalowsky et al., 2018; O’Brian et al., 2017; Takechi et al., 2012; Thiruchselvam et al., 2012), and seven included only Alzheimer’s disease (Edwards & Morris, 2007; Knapp et al., 2016; Nourhashemi et al., 2005; Soto et al., 2015; Wattmo et al., 2011, 2014; Webber et al., 1994; Tuokko et al., 1999). Two studies had no comparison group, meaning all participants had cognitive impairment and lived alone (Gibson & Richardson, 2017; Thiruchselvam et al., 2012). In 21 studies, the researchers compared PLACI and people with cognitive impairment living with others, without specifying who the “others” were (Alzheimer’s Association, 2012; Bass et al., 1992; Ebly et al., 1999; Edwards & Morris, 2007; Gage et al., 2015; Henderson et al., 2019; Kerpershoek et al., 2019; Knapp et al., 2016; Måvall & Malmberg, 2007; Michalowsky et al., 2016, 2018; Miranda-Castillo et al., 2010; Moholt et al., 2018; Nourhashemi et al., 2005; Rahja et al., 2018; Soto et al., 2015; Takechi et al., 2012; Wattmo et al., 2011; Webber et al., 1994). Six studies specificied the characteristics of the cohabiting person(s) (i.e., spouse, children; Ebly et al., 1999; Eichler et al., 2016; Schneider et al., 2002, 2003; Tuokko et al., 1999; Wattmo et al., 2014). Finally, two studies compared PLACI with people living alone without cognitive impairment (Huei-Ru et al., 2017; O’Brien et al., 2017), and one compared the proportion of people living alone in two groups of service users: one with cognitive impairment and one without cognitive impairment (Larsson et al., 2006). Fifteen studies recruited participants through specialist providers (memory clinics, dementia case managers, and research centers; Edwards & Morris, 2007; Henderson et al. 2019; Kerpershoek et al., 2019; Måvall & Malmberg, 2007; Miranda-Castillo et al., 2010; Moholt et al., 2018; Nourhashemi et al., 2005; Rahja et al., 2018; Schneider et al., 2002, 2003; Soto et al. 2015; Takechi et al., 2012; Wattmo et al., 2011, 2014; Webber et al., 1994), nine recruited through generalist local providers (home care or primary care; Bass et al., 1992; Chi et al., 1995; Eichler et al., 2016; Gage et al., 2015; Knapp et al., 2016; Michalowsky et al., 2016b, 2018; O’Brien et al., 2017; Thiruchselvam et al., 2012), four studies used national data sets (Alzheimer’s Association, 2012; Ebly et al., 1999; Gibson & Richardson, 2017; Tuokko et al., 1999), and four used local data sets (Edwards & Morris, 2007; Huei-Ru, 2017; Larsson et al., 2004, 2006). Sixteen studies reported only on social services (Gibson & Richardson, 2017; Huei-Ru et al., 2017; Kerpershoek et al., 2019; Larsson et al., 2004, 2006; Måvall & Malmberg, 2007; Michalowsky et al., 2018; Moholt et al., 2018; O’Brien et al., 2017; Rahja et al., 2018; Takechi et al., 2012; Thiruchselvam et al., 2012; Tuokko et al., 1999; Wattmo et al., 2011, 2014), six reported only on health services (Alzheimer’s Association, 2012; Bass et al., 1992; Chi et al., 1995; Knapp et al., 2016; Nourhashemi et al., 2005; Schneider et al., 2003), and 10 reported on both categories (Ebly et al., 1999; Edwards & Morris, 2007; Eichler et at., 2016; Gage et al., 2015; Henderson et al., 2019; Michalowsky et al., 2018; Miranda-Castillo et al., 2010; Schneider et al., 2002; Soto et al., 2015; Webber et al., 1994).

### Results of Individual Studies

We divided the results into two groups: health services and social services. Health services include inpatient (hospitalization), outpatient (primary care and specialist care), and home health services. Social services include home care, case management, and group settings (adult day care, congregate meals, and respite care). We also reported statistics on PLACI not accessing any services.

#### No formal services

Many PLACI did not receive any services. Three studies conducted in North America reported percentages on PLACI who did not receive any services. In the United States, a study of African Americans with dementia (Edwards & Morris, 2007) reported that 66% of those living alone and 64% of those living with others did not receive any services. An older study of people with Alzheimer’s disease in the United States found that those living alone were significantly more likely to use no services at all (14% vs 10%) (Webber et al., 1994). Furthermore, an older Canadian study by Tuokko et al. (1999) reported that 33% of PLACI did not use any services.

#### Health services

Half of the 32 included studies reported on use of health services. Most differentiated between specific types of services, but two studies reported on only overall use of hospital services, without specifying whether study participants exclusively received inpatient services. A recent British study reported no difference in the use of combined hospital services for people living alone with dementia compared to those living with others (Bieber et al., 2019; Henderson et al., 2019). An older study conducted in the United States (Webber et al., 1994) found that fewer people with Alzheimer’s disease living alone used hospital services compared to those living with others.

##### Inpatient services.—

Compared to people with cognitive impairment living with others, PLACI had higher hospitalization rates in three studies conducted in France, the United Kingdom, and United States (Soto et al., 2015), lower rates in one German study (Eichler et al., 2016), and similar rates in three studies from Canada, France, and United Kingdom (Ebly et al., 1999; Eichler et al., 2016; Henderson et al., 2019; Nourhashemi et al., 2005). With regard to length of stay, two recent studies from the United Kingdom and Germany reported no significant differences among PLACI and their counterparts living with others (Eichler et al., 2015; Gage et al., 2015). However, an older British study reported significantly longer hospital stays among PLACI (Chi et al., 1995).

##### Outpatient services.—

Three of six studies reported significantly lower rates among PLACI, with no studies identifying higher use of services by this population. A German study (Eichler et al., 2016) reported that PLACI were less likely to have visited a neurologist or psychiatrist than their counterparts living with others, although all had visited a primary care physician. In a recent British study, Henderson et al. (2019) found no difference in overall use of primary care services or mental health services between PLACI and those living with others. Another British study by Miranda-Castillo et al. (2010) found that the most common services used by PLACI were outpatient (60%), psychiatrists (55%), and primary care physicians (53%). An older British study, Schneider et al. (2002), found that PLACI were less likely to have seen a primary care physician (general practitioner), general outpatient service, or a psychiatrist. In the United States, a study of African American older adults with dementia found no difference in the overall use of primary care services between those living alone and those living with others. In an older study conducted in the United States, Webber et al. (1994) reported that fewer PLACI used physician services than those living with others.

##### Home health services.—

Overall, the percentages of PLACI using home health services varied widely by country. In France, a recent study (Soto et al., 2015) found no significant difference related to living arrangements: 88% of PLACI used home health services compared to 86% of their counterparts living with others. These rates were lower in an older French study reporting that 75% of PLACI used home health services, whereas 40% of their counterparts living with others did (Nourhashemi et al., 2005). A study from Germany reported that 56% of PLACI received professional help with medication compared to 20% of those living with others (Eichler et al., 2016). A British study reported no differences between PLACI and those with a coresident caregiver in accessing community psychiatry nurses (Schneider et al., 2002). The use of home health services was much lower in the United States, where a study of African Americans with dementia reported that only 10% of participants living alone accessed such services compared to 17% of those living with others (Edwards & Morris, 2007). An older U.S. study also found low rates of home health service use among older adults with Alzheimer’s disease, with only 3% of those living alone using these services (Webber et al., 1994). Finally, an even earlier study of U.S. public home health agency clients with cognitive impairment noted the lower use of services among those living alone versus those living with others (Bass et al., 1992).

#### Health service costs

The findings related to health service costs were mixed, with the most recent studies reporting no differences in costs. Three recent studies conducted in the United Kingdom and Germany found no differences in health service costs between PLACI and those living with others (Henderson et al., 2019; Knapp et al., 2016; Michalowsky et al., 2018). A British study (Gage et al., 2015) found that median total hospital costs over 6 months for PLACI were slightly higher (£15,120) than those living with others (£14,405). An older British study found significantly lower mean weekly costs for PLACI versus those living with a caregiver for medication, outpatient care, primary care, and public health services, but slightly higher costs for community health care (Schneider et al., 2003). Finally, in the United States, a report by the Alzheimer’s Association (2012) showed that PLACI had higher costs for outpatient health care and home health care but lower costs for inpatient hospital services, prescription medications, and hospice care; total annual health care costs were similar for PLACI and those living with others.

#### Social services

Of the 32 included studies, 26 reported on the use of social services.

##### Home care services.—

International comparisons highlighted that PLACI tend to access these services more than those living with others, and there is great variance in the use of essential home care services among countries, with the United States reporting lower rates. Specifically, a recent national study in the United States found that only 7% of PLACI had a paid assistant while 5% had another kind of paid staff, leaving the majority of PLACI without formal support (Gibson & Richardson, 2017). In contrast, a recent French study reported much higher utilization; 64% of PLACI used home care services compared to 47% of those living with others (Soto et al., 2015). In the United Kingdom, Henderson et al. (2019) reported that PLACI were nearly twice as likely as those living with others to use home care. Similarly, one British study by Gage et al. (2015) found that PLACI had larger social care packages (a higher use of formal services) than those living with others. Another British study (Miranda-Castillo et al., 2010) reported that PLACI received significantly more formal services than those living with others. In contrast, one British study, as well as an Irish study (O’Brien et al., 2019), reported similar levels of home care use between PLACI and those living with others (O’Brien et al., 2017; Schneider et al., 2002). The pattern of higher home care use among PLACI was replicated in other studies from Europe and Japan. In Norway, living alone predicted the use of home care services by people with cognitive impairment (Moholt et al., 2018). Furthermore, a recent European study found that living alone with dementia at baseline significantly predicted the use of home care over time (Kerpershoek et al., 2019). In Japan, living alone was an independent predictor of the need for public long-term care services for people with cognitive impairment (Takechi et al., 2012). Another Japanese study of enrollees in public long-term care services found that PLACI had a higher risk of increasing care needs (Huei-Ru et al., 2017).

##### Home help and personal care.—

Out of 12 studies, most (*n* = 9) reported higher use among PLACI, although overall many PLACI were reportedly not to receive these services. International comparisons, once again, indicated great variance. In Europe, home help use by PLACI ranged from 87% to 37%. In Germany, 87% of PLACI used professional home care services compared to 34% of those living with others (Eichler et al., 2016). In a British study, 45% of PLACI received home care, whereas only 12% of those living with others did (Miranda-Castillo et al., 2010). Another study conducted in the United Kingdom reported that home help services were used by 38% of PLACI but only 19% of people living with caregivers; meanwhile, personal care services were used by 59% of PLACI and 41% of people living with caregivers (Gage et al., 2015). In Sweden, 37% of PLACI used home help services compared to only 5% of counterparts living with family (Wattmo et al., 2014), although an older Swedish study reported higher percentages: 82% for PLACI versus 24% for those living with others (Måvall & Malmberg, 2007). In North America, lower home care use was reported in the United States among African Americans with dementia, with 26% of those living alone receiving “personal care/chore services” compared to 17% of those living with others (Edwards & Morris, 2007). In Canada, an older study by Tuokko et al. (1999) found that 20% of PLACI used home help, compared to 17% of those living with others.

##### Housekeeping/homemaker.—

In Canada, three older studies found that 50%–60% of PLACI used housekeeping services (Ebly et al., 1999; Tuokko et al., 1999). A lower use of housekeeping was reported in both Germany and the United States. In Germany, 14% of PLACI were reported to use these services versus 9% of those living with others (Eichler et al., 2016). Meanwhile, an older study conducted in the United States reported that 21% of PLACI had “homemaker chore” services (Webber et al., 1994).

##### Home-delivered meals.—

Overall, the use of home-delivered meals was low. Across Europe and North America, almost all studies found that around a quarter of PLACI used home-delivered meal services, compared to a significantly lower percentage of those living with others. In Germany, 26% of PLACI received in-home meals but only 12% of those living with others (Eichler et al., 2016). In the United Kingdom, 26% of PLACI received meals-on-wheels compared to 1% of those living with others (Miranda-Castillo et al., 2010). An older British study of people with dementia also found that living alone rather than with a caregiver was associated with a higher use of meals-on-wheels (Schneider et al., 2002). In a U.S. study of African Americans with dementia, 24% of those living alone received home-delivered meals versus 16% of those living with others (Edwards & Morris, 2007). An older U.S. study of people with Alzheimer’s disease with a predominantly White sample also found that one quarter (24%) of those living alone received the service (Webber et al., 1994). Three older Canadian studies, all focusing on people with dementia, reported similar findings. Tuokko et al. (1999) reported on two studies; in the first, 28% of participants living alone received home-delivered meals versus 5% of those living with others, whereas in the second, 16% of those living alone received meals compared to 4% of those with a spouse. Similarly, Ebly et al. (1999) found that one quarter (24%) of PLACI received home-delivered meals versus 4% of those living with others.

##### Day programs and congregate meals.—

Overall, low percentages were reported for these services. One German study of people with dementia found that 4% of those living alone attended day centers (Eichler et al., 2016). In Canada, two studies of people with dementia reported that 10% and 5% of those living alone in the respective samples attended day centers (Tuokko et al., 1999). In the United States, only 1% of African Americans with dementia living alone were found to attend day centers, with 11% using congregate meals (Edwards & Morris, 2007). An older study in the United States involving predominantly White people with Alzheimer’s disease reported that 11% of those living alone used senior centers (Webber et al., 1994).

##### Case management and social work.—

Rates about these services have been rarely reported, and the results showed limited access. In Australia, a recent study reviewing occupational therapy records for people with dementia found that case management was more commonly used for participants who lived alone (Rahja et al., 2018). In the United States, a study of African Americans with dementia reported that 28% of those living alone had a caseworker and 10% had a social worker; both rates were similar for those living with others (Edwards & Morris, 2007). An older U.S. study of predominantly White people with Alzheimer’s disease reported that 14% of those living alone received case management versus 4% of those living with others (Webber et al., 1994). A British study reported no differences between PLACI and those who had a coresident caregiver in accessing social work services (Schneider et al., 2002).

#### Social service costs

Five studies reported on costs of social service use, providing mixed findings. In Germany, Michalowsky et al. (2016b) found that PLACI had significantly higher annual costs for home care, and a more recent study from the same group (2018) found PLACI had three times higher annual costs for home care and short-term institutional care (combined) than people living with others. In the United Kingdom, a recent study (Henderson et al., 2019) reported no significant difference in paid care costs when comparing PLACI with those living with others. Another British study reported that mean costs of social care services for PLACI were more than twice as high as for people living with a caregiver (Gage et al., 2015). Finally, an older British study found no differences in costs for day care, social services, or respite services between PLACI and those living with others (Schneider et al., 2003).

## Discussion

In this first systematic review of the use of formal health and social services among PLACI worldwide, we identified wide variability among countries and major gaps in service use that are likely to affect the health and well-being of this population. Results for the use of health services were mixed, although our findings suggest that PLACI may have fewer physician visits than counterparts living with others. We also found that overall home health use was higher in Europe than in the United States, with 75%–88% of PLACI in France and 56% in Germany using home services compared to only 3%–10% in the United States. The use of social services was generally higher among PLACI compared to those living with others. However, a large proportion of PLACI did not use these supportive services, again with particularly low rates reported in the United States. Data on costs of health and social services were heterogeneous, showing no clear trend. Studies based in North America reported that large portions of PLACI do not access any services.

Our findings expand on the conclusions of a systematic review by Weber et al. (2011), which showed that people with dementia frequently use medical services and generally have regular visits with physicians. Specifically, we found that PLACI may not visit physicians as often as those who live with others. Weber et al. (2011) also reported lower community service use among people with dementia, compared to those without dementia. This is consistent with our results, which suggest that people with dementia use limited social services. Our review further indicates that PLACI appear to use community services more than those living with others, albeit still at low rates. In addition, our findings confirm Bieber et al.’s (2019) estimate that government policies and system-level differences influence the use of formal services among PLACI. In their scoping review, Bieber’s team found fewer disparities between PLACI and those who live with others in countries where supportive services are publicly funded and universally available, like in Germany. To show the influence of social policies supporting PLACI, the authors contrasted the United States with Sweden: whereas U.S. dementia policies are “designed to meet the needs of older adults with family support” (p. 11), Swedish policies are centered on the needs of the person with cognitive impairment, independent of their living arrangement or support system. A major contribution of our study is that it expands these estimates because we reported on access to services that are essential to PLACI (e.g., home care aides) in several countries, with countries with more robust social policies (e.g., Germany, France, the United Kingdom) providing subsidized home care aides to larger percentages of PLACI than the United States.

Indeed, the varying policies, as well as differing state budgets for these services in different countries may have affected our findings. Furthermore, barriers to accessing and using services, in addition to policy and practice implications for improving access to care to PLACI, likely vary by country. For example, in Germany, a country with public universal long-term care insurance (Campbell et al., 2010), PLACI’s low rate of hospitalization may be explained by the presence of a strong support system in their homes (Michalowsky et al., 2019). This strong support system might also lead to the conclusion of Eichler et al. (2016) that PLACI “did not seem to be at an increased risk for their health, even if they lacked the support of an informal caregiver” (p. 628). Yet, a barrier to the use of home services is the low rate of timely dementia diagnoses (Eichler et al., 2015; Michalowsky, 2020; Michalowsky et al., 2016a). On the other hand, promising practices to improve the care of PLACI include innovative systems to better coordinate dementia care from different providers (Michalowsky et al., 2019; Thyrian et al., 2017). In France, a country with broad, redistributive, and compulsory social insurance (Nay et al., 2016), PLACI receive long-term services and supports funded by a national disability fund by subregional entities. Access to social services depends on the assessment of functional disability, need for services (related to living arrangements and availability of caregivers), and income (Rapp et al., 2015). Barriers to access to social services for PLACI include a limited generosity of the subregional entities due to constrained budgets, which are sometimes unable to fund sufficient services and equipment to address all PLACI’s needs (Rapp et al., 2015). In addition, France, as well as other Organisation for Economic Co-operation and Development (OECD) countries, is facing a shortage of home care aides, which is considered an unattractive career because of its low pay, burden, and limited training (OECD, 2020). Furthermore, as in most OECD countries, French medical and social services for PLACI are fragmented and poorly coordinated (OECD, 2018). In the United Kingdom, the tax-funded National Health System was created in 1948 to provide access to health care for all based on need, and not the ability to pay (Hellowell & Ralston, 2015). In addition, local authority social service departments provide home care and other support which is based on an assessment of personal means and needs. As a result, PLACI have access to health care that is free at the point of delivery. They will receive support from social services, provided that their needs are sufficiently great and their local authority has sufficient funds (Glendinning, 2017). Local authorities may vary the threshold at which they can provide support due to budget limitations, and all but the PLACI with the lowest means may have to contribute part or all of the cost of the social care services they receive. Pathways to dementia services in the United Kingdom have been reported as being difficult to navigate overall (Peel & Harding, 2014; Rothera et al., 2008), but a national dementia strategy over the last decade has provided additional funding and service reconfigurations designed to improve the situation. Also in Canada, a country with universal health insurance, the provision of social services mostly relies on local rules and budgets because the provisions of health care services are regulated at provincial level. As a result, some of the 13 provinces may provide greater home services than other ones. According to the Canadian Academy of Health Science (2019), fragmentation in the payment and provision of social services is a major barrier to the proper care of PLACI. Canadian providers of care have reported their difficulties in properly supporting PLACI (De Witt & Ploeg, 2016). Other issues include shortages of personal care workers for PLACI, as well as low wages with limited benefits (Public Health Agency of Canada, 2019).

This review has several limitations. Due to the heterogeneity of the study contexts and measures, we were not able to conduct meta-analyses. Furthermore, we only included studies published in English, limiting the range of countries represented. Finally, the data did not allow us to establish the level of need for services for PLACI, or to consider associated outcomes. We were unable to identify subgroups, such as those who live alone because they are less impaired or well supported, and those who are struggling with unmet needs.

Six major implications can be drawn from this study. First, its international comparisons highlighted different levels of service use by country, thus pointing to the crucial role of social policies and state budgets to support PLACI. These comparisons encourage researchers to better understand the influence of state policies on their findings and when reporting findings per country from systematic reviews of international literature. At the policy level, national dementia plans must focus on those living alone, which is seldom the case. Specifically, social policies that provide privilege access to essential services and supports to PLACI should be enforced. Second, although our review quantifies the use of various types of services, we have limited information about the quality or appropriateness of these services. For example, it is not clear whether hospitalization represents appropriate acute care or inadequate primary care. More research is needed to understand and measure the appropriateness and cultural sensitivity of formal services accessed by PLACI. Third, almost half of the reviewed studies recruited their samples through specialist providers, such as memory clinics. This is unsurprising given the challenges of recruiting PLACI (Portacolone et al., 2017; Soniat, 2004). However, such strategies are likely to exclude the majority of PLACI who do not receive specialist care services and may have more unmet needs. As a result, special efforts should be made by researchers to recruit participants from the community rather than memory clinics. Fourth, none of the included studies reported the use of emergency services, although high levels of emergency service use may be a marker of inadequate supportive care for PLACI (LaMantia et al., 2016; Rosenwax et al., 2015). Thus, more research on the use of emergency services among PLACI is needed. 

As this study marks the first systematic literature review published on PLACI, to further our understanding of this population, future systematic reviews should focus on qualitative studies about PLACI, interventions to support this population, and both outcomes and risks. In this review, the only methodological issue that we encountered was the agreement on the categorization of services, which was reached by consensus. Finally, the included studies generally neglected to characterize the race and ethnicity of participants; when they included such information, racial and ethnic minorities were underrepresented. Such underrepresentation is concerning, especially considering that Black and Latinx people are, respectively, 2 and 1.5 times more likely to develop cognitive impairment than White people (Mayeda et al., 2016). Indeed, the only U.S.-based study in our review focusing on African American PLACI (Edwards & Morris, 2007) reported much lower use of social services than the other studies. Immigration status also has a multifaceted relationship with dementia risk (Schmachtenberg et al., 2020; Xu et al., 2017). With the exception of studies from our group (Portacolone et al., 2021), we have little understanding of the needs of immigrants with cognitive impairment who live alone. It is thus crucial to conduct research that represents the growing diversity of PLACI worldwide.

## Conclusion

People with cognitive impairment need access to both health and social services to maintain their health and well-being. For those who live alone, our review highlights gaps in the access to and use of services compared to those who live with others. Surprisingly, even the presence of social policies that offer access to essential services is not a guarantee that these services will be used because of barriers due to limited state funding, shortage of care workers, or the lack of diagnosis or informal caregivers. In the United States, most barriers are compounded by the fact that essential long-term services and supports are only available to low-income PLACI. In light of these considerations, more research on specific barriers to the use of essential services among PLACI is needed. To promote future research on this population, it is critical to improve our ability to identify PLACI. For example, it is essential that both clinicians and researchers ask about living arrangements. We also encourage a greater focus on living arrangements in national dementia care plans and dementia care models to better support those who live alone.

## Supplementary Material

igab004_suppl_Supplementary_Materials
