# Supplementary material for: Use of Services by People Living Alone With Cognitive Impairment: A Systematic Review
Source: Innov Aging. 2021 Jan 18;5(1):igab004. doi: 10.1093/geroni/igab004 (PMC7990060; doi:10.1093/geroni/igab004)
Supplement: igab004_suppl_Supplementary_Materials [file igab004_suppl_supplementary_materials.docx]

**SUPPLEMENTARY MATERIAL**

**TABLE 1. OLDER ADULTS LIVING ALONE, SELECTED COUNTRIES^A^**

| **Area** | **Country** | **Percentage of households living arrangements of persons aged 60 and over** | | | **Number of persons aged 60 and over** |
| --- | --- | --- | --- | --- | --- |
|  |  | **Alone** | **With Spouse only** | **With Children** |  |
| Africa | Egypt | 13.5 | 24.4 | 59.0 | 6,337,500 |
|  | Nigeria | 12.0 | 8.9 | 60.6 | 7,202,400 |
|  | South Africa | 11.2 | 15.3 | 47.0 | 3,701,500 |
| Asia | China | 8.2 | 25.0 | 57.4 | 171,120, 400 |
|  | India | 4.9 | 15.5 | 71.0 | 96,137,100 |
|  | Iran | 14.4 | 25.5 | 56.8 | 5,361,200 |
|  | Japan | 15.5 | 35.8 |  | 38,941,200 |
|  | Philippines | 5.4 | 10.9 | 65.2 | 6,115,100 |
|  | Thailand | 6.0 | 12.8 | 68.4 | 8,703,500 |
| Europe | Austria | 30 | 45.9 | 20.5 | 1956800 |
|  | Belgium | 25.1 | 52.5 | 15.8 | 2539500 |
|  | Finland | 32.9 | 54.00 | .. | 1,328,000 |
|  | France | 31.2 | 56.00 | 10.2 | 14,552,000 |
|  | Germany | 31.1 | 59.0 | 6.9 | 21,082,400 |
|  | Italy | 28.5 | 41.0 | 27.4 | 16,042,900 |
|  | Netherlands | 31.1 | 54.8 | .. | 3,664,900 |
|  | Poland | 22.1 | 38.9 | 33.8 | 7,416,600 |
|  | Spain | 17.3 | 41.4 | 33.5 | 10,502,700 |
|  | Sweden | 27.9 | .. | .. | 2,334,900 |
|  | UK | 32.4 | 53.6 | 10.9 | 14,344,500 |
| North America | Canada | 24.1 | 50.1 | 18.0 | 6,819,400 |
|  | United States | 25.1 | 46.3 | 19.4 | 56,707,200 |
| South America | Argentina | 18.6 | 37.9 | .. | 5,931,400 |
|  | Brazil | 13.3 | 21.8 | 35.1 | 19,743,700 |
|  | Mexico | 11.3 | 20.6 | 55.4 | 9,853,100 |
|  | Peru | 13.1 | 18.9 | 55.1 | 2,627,900 |
|  | Venenzuela | 7.7 | 8.5 | 64.8 | 2,399,100 |
| Oceania | Australia | 23.2 | 51.5 | .. | 4,190,200 |
|  | New Zealand | 24.4 | 53.9 | .. | 802,500 |

^a^ Data from the United Nations, Living arrangements of older persons: a report on an expanded international dataset, 2017.

**TABLE 2. DETAILED CHARACTERISTICS OF INCLUDED STUDIES**

| **First author, year, country** | **Design/**  **methodology** | **Sample *(total n and type of cognitive impairment, race/ethnicity, gender, living alone, age,)*** | **~~Setting~~ Recruitment strategy** | **Comparison group** | **Results** |
| --- | --- | --- | --- | --- | --- |
| **HEALTH SERVICES** | | | | | |
| Henderson, 2019, UK | Cross-sectional (baseline from self and proxy report data from cohort study) | 1547 PWD^[[1]](#footnote-1)^. (Mild-to-moderate dementia). Race/ethnicity NR^[[2]](#footnote-2)^. 56% male, 44% female. 20% living alone. Mean age 76.4 (sd 8.6). | Recruited from NHS clinics and research portals in England, Scotland, Wales | All had dementia, compared living alone *vs.* not | No difference in use or costs for primary care or mental health. No difference in use or costs for hospital services, medications, or equipment. No difference in total paid care costs. All findings over previous 3 months. |
| Michalowsky, 2018,  Germany | Cross-sectional study (data from self and proxy report) | 425 PWD (None-to-severe cognitive impairment and formal dementia diagnosis). Race/ethnicity NR. 57% female. 48.7% lived alone. Mean age 80.2 (sd 5.3). | Baseline data from cluster-RCT GP-based intervention trial | All had CI^[[3]](#footnote-3)^, compared living alone *vs.* not | In multivariate model, no association between living alone and medical care costs. |
| Eichler, 2016,  Germany | Cross-sectional (analysis of self and proxy report, at baseline of RCT) | 511 community dwelling PWD (any type of dementia). Race/ethnicity NR, 59.3% female, 50.9% living alone, mean age 80.3 (sd 5.5). | GP-based RCT of person-centered intervention in Mecklenburg-Western Pomerania | All had dementia, compared living alone *vs.* not and informal caregiver *vs.* no informal caregiver | Fewer people living alone had visited neurologist/psychiatrist (p=0.032) and had been admitted to hospital (p=0.022). No significant difference in number of GP visits (all had visited GP at least once) or days in hospital. No statistically significant differences in use of health care services between those with and without informal caregiver, within live alone group. More people living alone used professional help with medications (p<0.001). |
| Knapp, 2016, UK | Retrospective cohort study Data collected from medical records) | 3075 community-dwelling PWD (AD diagnosis). 82% white, 10.1% Caribbean/African, 3.1% East/South Asian, 4.6% mixed/unknown. 67% female, 33% male. 26% living alone. Mean age NR. 1.4% 40-59 years, 6.7% 60-69 years, 34.1% 70-79 years, 48.3% 80-89 years, 9.4% 90+ years. | Records of secondary mental healthcare provider ~~in London~~ | All had AD^[[4]](#footnote-4)^, compared living alone *vs.* not | People living alone had higher odds of general inpatient admission in 6 months OR 1.26 (95% CI 1.05-1.49, p=0.01), and of mental health inpatient admission in 6 months OR 2.56 (95% CI 1.76-3.71, p<0.01). Costs not significantly different for living alone vs living with others (p=0.18 for general hospital admission, p=0.44 for mental health inpatient admission). |
| Gage, 2015,  UK | Cohort study with 6- and 12-month follow up (data from self and proxy report) | 95 PWD (any type of dementia) living in domiciliary situation (full sample of 109 people included those in care homes). Race/ethnicity NR, 66.3% living alone. Living alone: 79.4% female, mean age 85.9 (sd 5.48)^[[5]](#footnote-5)^ | Recruited through inpatient hospital liaison psychiatry in central England | All had dementia, compared living alone at home *vs.* not alone. | No difference in length of index hospital stay (p=0.844). Median total hospital costs (inpatient + outpatient, day hospital, ED) over 6 months prior to baseline assessment: £15,120 (interquartile range £10,576-21,960) for people living alone and £14,405 (IQR £11,024-24,642) for people living with others, no significance data reported. |
| Soto, 2015, France | Prospective cohort study over 24 months (data collected from self and proxy report) | 1131 PWD (mild-to-moderate AD). Race/ethnicity NR. 30.8% living alone. Living alone: 90.2% female. Mean age 81.1 (sd 5.2).  Living with others: 59.1% female. Mean age 79 (sd 5.8). | Memory clinics in France. Data from cluster randomized trial of memory clinic intervention. | All had AD, compared those living alone *vs.* not. | People living alone at inclusion were more likely to be hospitalized (33.3%) over the 2 years of follow-up than those living with others (30.0%); significance not reported. Incidence of hospitalization: 31.2 per 100 person-years for living alone (95% CI = 25.5–36.9), 25.6 per 100 person-years for living with others (95% CI = 22.3– 28.8). Cox proportional hazards models: living alone was independent predictor of hospitalization (HR = 1.33, 95% CI = 1.01–1.74). No difference between groups in use of home healthcare services (p=0.24). |
| Alzheimer’s Association, 2012, US | Cross-sectional study (data collected from self and proxy reports, as well as medical records) | Any dementia diagnosis. Of all with dementia diagnosis, 15% living alone. Of those living in the community, 25% living alone. No other demographics reported. | Medicare Current Beneficiary Survey for 2008 | All had dementia, compared people living alone and not living alone. | People living alone with dementia had similar total health care costs as those not living alone, with higher costs for outpatient health care institutions and home health care and lower costs for inpatient hospital services, prescription medications and hospice care. Per-person payment per year for people living alone: inpatient hospital $7377, medical care provider $7015, home health $2162, hospice $418, prescriptions $3,671, Other health care institutions $3851, total $25,389. Living with others: inpatient hospital $9782, medical care provider $6794, home health $1327, hospice $1285, prescriptions $4,638, other health care institutions $1219, total $25,943. For every 1000 people who lived alone with dementia, there were 744 hospital stays vs 705 hospital stays if not living alone. No significance data reported. |
| Miranda-Castillo, 2010, UK | Cross-sectional survey (data collected from self and proxy report) | 152 PWD (any dementia). Race/ethnicity NR. Living alone: 76% female. Mean age 81.7 (sd 5.9). Living with others: 39.2% female. Mean age 78 (sd 6.9). | Recruited through service providers (85% through NHS) | All had dementia, compared living alone *vs.* not. | Most common services used by people with dementia living alone: outpatient (60.0%), psychiatrist sessions (55.2%), general practitioner appointments (53.3%) (comparison/significance NR). |
| Edwards, 2007,  US | Cross-sectional (data collected from self and proxy report) | 343 PWD (diagnosis of possible or probable AD). Race/ethnicity 100% African American, 52% living alone. Living alone: 87% female, mean age 81.3 (sd 9.3). Living with others: 67% female, mean age 80.4 (sd 7.4). | Provider of outreach, diagnostic, and treatment to minority and low-income persons in metropolitan area | All had AD, compared living alone *vs.* with others | 78% of those living with others and 75% of those living alone had seen physician in last 12 months – lower than in large national samples (significance NR). No significant difference in home health/home health aide. |
| Nourhashemi, 2005, France | Cohort study (data collected from self and proxy report) | 677 community-dwelling people (AD diagnosis), 28% lived alone. Race/ethnicity NR. Living alone: 6.46% male. Mean age 79.4 (sd 5.9). Living with others: 38.28% male. Mean age 76.9 (sd 7.0). | Recruited through study centers of French AD Network | All had AD, compared those living alone *vs.* not. | Hospitalization rates over one year follow up were not different between living alone and not (p=0.727). People living alone used home health services more frequently (p=0.0217). |
| Schneider, 2003, UK | Longitudinal study, over 17 months (data collected from self and proxy report) | 132 PWD (moderate-to-severe dementia). 93% white, others NR. 64% female. Mean age 81 (sd 7.7). | Recruited from community psycho-geriatric service caseloads of people living in South London | All had dementia, compared those living alone with those with co-resident carer. | At Time 1, people living alone had lower costs for medication, outpatient care, primary care and consequently for NHS services overall. The greater use of NHS services by people with co-resident caregivers over people living alone was highly significant at Time 1, but not at Times 2 or 3. At Time 1, people living alone had slightly higher costs for community health care (for example, community psychiatric nursing); this was marginally statistically significant (independent means t=1.9, p=0.051; Mann–Whitney U=852, p=0.016). |
| Schneider, 2002, UK | Cross-sectional (baseline of longitudinal study; data collected from self and proxy report) | 132 PWD (moderate-to-severe dementia). 93% white, others NR. 64% female. Mean age 81 (sd 7.7). | Secondary care caseloads (i.e. specialist dementia services) in South London | All had dementia, compared those living alone and those with co-resident caregiver | People living with caregiver received more services overall than those living alone (mean 7.8 vs. 6.4, p=0.01). People living alone less likely to have used psychiatrist, GP, outpatient care or overall community health care. Overall health and social care services: no difference between living alone and with co-resident caregiver. No difference in inpatient services, psychologist, community psychiatric nurse, primary care nurse, primary care OT, primary care PT, primary care chiropodist. |
| Ebly, 1999,  Canada | Cohort study with follow-up at two years (data collected from self and proxy report) | 317 PWD (any dementia diagnosis). Race/ethnicity NR, 31.5% living alone. Live alone: 77% female, mean age 82.9 (sd 5.5). Not alone: 53.9% female, mean age 81.7 (sd 6.2). | National study | All had dementia. Compared living alone *vs.* not alone | No significant difference in overnight hospitalization (live alone 28.3%, not alone 29.5%, p=0.87) |
| Chi, 1995,  UK | Retrospective cohort analysis over 4 years (data collected from medical records) | 144 elderly people (any cognitive impairment). Race/ethnicity NR, 65% female, 46% living alone, age range 75-97. | Cohort of elderly people with different levels of cognitive and physical frailty | Compared CI *vs.* physically frail *vs.* physically healthy groups; living alone *vs.* not | Living alone predicted longer hospital stays for cognitively impaired group (median 28.5 vs 0 days, p=0.002). 79% of people living alone with cognitive impairment were admitted to geriatric wards, compared to 32% of physically frail and 33% of physically healthy group (p=0.009). |
| Webber,1994,  US | Cross-sectional (data collected from proxy report and medical records) | 2505 treatment center clients (diagnosis of possible or probable AD). 19.1% living alone. Living alone: 81% white, 1.3% Asian, 7.5% Black, 8.4% Hispanic, 84.1% female, 18.4% under 65, 22.6% age 65-74, 50.2% 75-84, 8.8% 85 and over.  Living with others: 78.4% white, 2.8% Asian, 8.4% Black, 8.4% Hispanic, 63.6% female, 20.7% under 65, 29.3% 65-74, 41.3% 75-84, 8.7% 85 and over. | University-based Alzheimer’s Disease Diagnostic and Treatment Centers in California | All had AD, compared living alone *vs.* living with others. | Fewer people living alone used physician services (p<0.0005), hospital services (not otherwise specified) (p=0.01), and adult day health (p<0.0005). In multivariate models, living arrangement (alone vs with others) was most consistently significant predictor of service use – physician (OR 0.64, CI 0.51-0.79), hospital (OR 0.65, CI 0.48-0.89), adult day health care (OR 0.45, CI 0.28-0.74). No difference in home health care. People living alone also more likely to use no services (p=0.14). |
| Bass, 1992  US | Cross-sectional (data collected from self report and medical records) | 343 clients (any cognitive impairment). Home health users: 52% white^[[6]](#footnote-6)^, 78% female, 35% living alone, mean age 81.74 (sd 9.06). Social service users: 54% white, 76% female, 53% living alone, mean age 78.39 (sd 8.33) | Medicare/mental health certified home health provider in Ohio; people who used community services, had case manager | Compared living alone *vs.* with others at different levels of CI | For those with higher than average levels of cognitive impairment, living with another person predicted use of more home health service hours (unstandardized regression coefficient 2.56). |

| **SOCIAL SERVICES** | | | | | |
| --- | --- | --- | --- | --- | --- |
| Henderson, 2019, UK | Cross-sectional (baseline from self and proxy report data from cohort study) | 1547 PWD^[[7]](#footnote-7)^. (Mild-to-moderate dementia). Race/ethnicity NR^[[8]](#footnote-8)^. 56% male, 44% female. 20% living alone. Mean age 76.4 (sd 8.6). | Recruited from NHS clinics and research portals in England, Scotland, and Wales | All had dementia, compared living alone *vs.* not | People living alone were nearly twice as likely as those living with others to use social care (two-part model OR 1.882 (95% CI 1.274, 2.780, p<0.01)). No difference in use of day services. No difference in costs for social care, or day services. No difference in total paid care costs. |
| Kerpershoek, 8 European countries (Germany, the Netherlands, Sweden, Norway, Ireland, UK, Portugal, Italy), 2019 | Cohort study, with measurements at baseline, 6, and 12 months (data collected from self and proxy report data from cohort study) | 451 community-dwelling people with mild to moderate dementia. Race/ethnicity NR. 54% female, 46% male. 28% living alone. Mean age 77.8 (sd 7.9) | Recruited from European prospective cohort study through: general practices, memory clinics, case managers, community mental health teams, ads | All had dementia, compared living alone *vs.* not alone | Living alone at baseline significantly predicted the use of home social care at T1 or T2, OR 0.44 (95% CI 0.23‐0.85, p<0.05; living alone coded as 0). Living alone at baseline significantly predicted the use of home personal care at T1 or T2  OR 0.29 (95% CI 0.16‐0.52, p<0.01; living alone coded as 0). For day care, living alone at baseline predicted use at T1 or T2  OR 0.51 (95% CI, 0.30‐0.87, p<0.05; living alone coded as 0) |
| Michalowsky, 2018,  Germany | Cross-sectional study (data from self and proxy report) | 425 PWD (None-to-severe cognitive impairment and formal dementia diagnosis). Race/ethnicity NR. 57% female. 48.7% lived alone. Mean age 80.2 (sd 5.3). | Baseline data from cluster-RCT GP-based intervention trial | All had CI, compared living alone *vs.* not. | Linear mixed regression models: from payer perspective, formal care costs significantly positively associated with living alone (b = 1325; p = 0.001). PWD who lived alone had three times higher formal care costs (living alone 2455 € versus not alone 822 €). |
| Moholt, 2018, Norway | Cross-sectional survey (data collected from proxy report) | N=430 (diagnosis of dementia or CI consistent with dementia). 89.8% Non-Sami, 5.3% Sami one marker, 4.9% Sami two markers. 63.5% female, 36.5% male. Living alone NR. Mean age 82.59 (sd 6.86). | Recruited caregivers who used community health services in 32 municipalities | All had dementia, compared living alone *vs.* not | Living alone predicted use of home-based services (beta -1.12 (95% CI (-1.34, -0.87), p<0.001; note that living alone was coded 0). |
| Rahja, 2018, Australia | Cross-sectional (audit of occupational therapy case notes) | N=87 (any dementia diagnosis or probable dementia). Race/ethnicity NR. 50.6% female, 49.4% male. 41.4% living alone. Mean age 81.8 (sd 9.77). | Case notes audited from four different service contexts | All had CI, compared living alone *vs.* not. | Case management (p=0.024) was more commonly used for participants who lived alone in the community. |
| Gibson, 2017,  US | Cross-sectional (data collected from self and proxy report, as well as medical records) | 710 PWD (possible or probable dementia) and CI (any CI). 69.9% white, 27.5% African American, 1.7% American Indian, 1.9% Asian American, 2.8% other race. 69.7% female, 30.3% male. 100% living alone. Mean age NR, 71.2% aged over 80. | National Health & Aging Trends Study (NHATS) first wave, Medicare enrollees aged 65+, subsample living alone with possible/probable dementia | All living alone with CI | 116 participants gave information about primary caregiver. 6.9% had a paid assistant. 5.2% had another kind of paid staff. Others were relatives; less than 1% friends. |
| Huei-Ru, 2017, Japan | Retrospective cohort study (data collected from insurance claims) | 23,638 PWD (any type of dementia) and 53,521 people without dementia. Race/ethnicity NR. 69% female. 18.2% living alone. Mean age 83.48 (sd 7.6). | Long-term care insurance claims database in Kyoto, including those aged 65+ | Compared living alone with dementia to others | Cox proportional hazard regression model: people with dementia living alone had a higher risk of care needs increases (HR: 1.09, 95% CI: 1.04–1.15). |
| O’Brien, 2017, Ireland | Cross-sectional audit (data collected from medical records) | 935 older people (diagnosis of dementia or suspected to have CI). Race/ethnicity NR. 65% female. 55% living alone. Mean age 83.7 (sd 7.4).^[[9]](#footnote-9)^ | All clients 65+ receiving publicly funded home care packages (5+ hours/week) in area of Dublin | All living alone, compared dementia *vs.* no dementia | No difference in % living alone between dementia and non-dementia groups (all receiving home care) (p=0.068). |
| Eichler, 2016,  Germany | Cross-sectional (analysis of self and proxy report, at baseline of RCT) | 511 community dwelling PWD (any type of dementia). Race/ethnicity NR, 59.3% female, 50.9% living alone, mean age 80.3 (sd 5.5). | GP-based RCT of person-centered intervention in Mecklenburg-Western Pomerania. | All had dementia, compared living alone *vs.* not and with/without informal caregiver | More people living alone used professional services, including home care (p=0.022) and home-delivered meals (p<0.001). No significant difference in use of professional day care or professional housekeeping. More people living alone without informal caregiver used professional housekeeping (p=0.003) – no other statistically significant comparisons. |
| Michalowsky, 2016, Germany | Cross-sectional study (data collected from self and proxy report, as well as medical records) | 262 PWD (any type of dementia). Race/ethnicity NR. 56.5% female. 54.2% lived alone. Mean age 80.69 (sd 5.4). | Baseline data from cluster-RCT GP-based intervention trial | All had dementia, compared living alone *vs.* not. | Multivariate logistic analysis: formal care costs significantly positively associated with living alone (p=0.005). Bivariate model: people with dementia who lived alone had significantly higher annual formal costs (2,504 € vs 920 € ) but significantly lower informal care costs (13,634 € vs 18,872 € ). |
| Gage, 2015,  UK | Cohort study with 6- and 12-month follow up (data from self and proxy report) | 95 PWD (any type of dementia) living in domiciliary situation (full sample of 109 people included those in care homes). Race/ethnicity NR, 66.3% living alone. Living alone: 79.4% female, mean age 85.9 (sd 5.48)^[[10]](#footnote-10)^ | Recruited through inpatient hospital liaison psychiatry in central England | All had dementia, compared living alone at home *vs.* not alone. | Personal care used by 59% of people living alone, 41% of people living with caregiver, 50% of people living with non-caregiver. Home help used by 38% of people living alone, 19% of people living with caregiver, 0% of people living with non-caregiver. Social care packages larger (i.e. more contacts with service) for those living alone vs. with others, significance NR. Mean cost of social care over 6 months: living alone £6774 (sd 7656), living with caregiver £3214 (sd 5399), living with non-caregiver £6312 (sd 5782), significance NR. Median community health service (GP, psychiatrist, nursing, allied professionals, social worker, continence supplies) costs over 6 months prior to baseline assessment: £284 (IQR £148-573) for living alone, £248 (IQR £185-498) for living with others. |
| Soto, 2015, France | Prospective cohort study over 24 months (data collected from self and proxy report) | 1131 PWD (mild-to-moderate AD). Race/ethnicity NR. 30.8% living alone. Living alone: 90.2% female. Mean age 81.1 (sd 5.2).  Living with others: 59.1% female. Mean age 79 (sd 5.8). | Memory clinics in France. Data from cluster randomized trial of memory clinic intervention | All had AD, compared living alone *vs.* not. | Significantly more people living alone used non-health home services (housekeeper, home-delivered meals, care attendant, tele-alarm system) (p<0.001). |
| Wattmo, 2014, Sweden | 3-year, prospective, open, nonrandomized, multicenter study (data collected from self report) | 1021 people with mild-to-moderate AD. Race/ethnicity NR. 35% living alone.  Living alone: 85% female. Mean age, baseline 77.1 (sd 6.4).  Living with others: 53% female. Mean age, baseline 74.2 (sd 7.1). | Recruited from 14 memory clinics located in diverse geographical parts of Sweden | All had AD, compared living alone and living with family member. | 37% of those living alone used home-help services at the start of ChEI therapy vs. 5% of those living with family (p<0.001). Mean ± SD usage of home-help services was 5.5±4.8 hours/week, no significant difference according to living status. After 3 years of treatment, 71% of those living alone used a mean of 9.2±6.6 hours of home-help services per week. |
| Takechi, 2012, Japan | Cross-sectional study (data collected from self and proxy report, as well as medical records) | 201 people with CI (any type of CI). Race/ethnicity NR. 65.7% female. 16.4% living alone. Mean age 78.7 (sd 7). | People attending outpatient memory clinic in Kyoto | All had CI, compared living alone *vs.* not. | Multiple regression: living alone was independent predictor of care rank (b: -0.18, p = 0.03). |
| Thiruchselvam, 2012, Canada | Prospective cohort study (data collected from self and proxy report) | 339 people with CI. Race/ethnicity NR. 79% female, 21% male. 100% living alone. Mean age for two subgroups: 83.77 (sd 6.58) and 84.28 (sd 6.03). | Sample drawn from larger prospective study, included people with CI taking at least one medication | All had CI and lived alone. | Number of areas in which formal help received for participants without nonadherence: mean 1.56 (sd 1.28); for participant with nonadherence 1.68 (sd 1.17). Significance levels NR. |
| Wattmo, 2011, Sweden | 3-year, open-label, observational, nonrandomized, multicenter study (data collected from self report) | 880 people with AD. Race/ethnicity NR. Admitted to nursing home: 72% female. 50% living alone. Mean age, baseline 76.4 (sd 6.2). Not admitted to nursing home: 60% female. 30% living alone. Mean age, baseline 74.7 (sd 7.2). | Recruited from 14 memory clinics located in diverse geographical parts of Sweden | All had AD, compared living alone *vs.* not. | People living alone received greater increase in home help service per week (p<.001). No significant difference was found regarding living status for increase in adult day care per week. |
| Miranda-Castillo, 2010, UK | Cross-sectional survey (data collected from self and proxy report) | 152 PWD (any type of dementia). Race/ethnicity NR. Living alone: 76% female. Mean age 81.7 (sd 5.9). Living with others: 39.2% female. Mean age 78 (sd 6.9). | Staff member of relevant services approached potential participants (85% through NHS facilities) | All had dementia, compared living alone *vs.* not. | People living alone received significantly more formal services (mean 8.8, sd 4.5) than those living with others (mean 6.9, sd 4.6) (p<0.05). Significantly more PWD living alone (45.7%) received help from home care worker, compared to those living with others (12%) (p<0.001). More of those living alone (26.5%) than with others (1.2%) used meals-on-wheels (p<0.001). |
| Edwards, 2007,  US | Cross-sectional (data collected from self and proxy report) | 343 PWD (diagnosis of possible or probable AD). Race/ethnicity 100% African American, 52% living alone. Living alone: 87% female, mean age 81.3 (sd 9.3). Living with others: 67% female, mean age 80.4 (sd 7.4). | Memory and Aging Project Satellite (provides outreach, diagnostic, and treatment to minority and low-income persons in St. Louis metropolitan area) | All had AD, compared living alone *vs.* living with others | Few received any formal services across whole sample. Living alone group received significantly more social services (p=0.0003), specifically congregate meals (11% vs 1%, p=0.001). No significant difference in home-delivered meals, personal care/chore service, transportation, respite/adult day care, social work. Logistic regression: living alone predicted formal service use (OR 1.98, CI 1.15-3.41). |
| Måvall, 2007,  Sweden | Cohort study (data collected from proxy report) | 51 caregivers of PWD (any type of dementia). Race/ethnicity NR. 59% female, 41% male. 43% living alone. Mean age 79 (sd NR). | Attendees of a dementia day care center for 12 months | All had dementia, compared living alone *vs.* not | At baseline: more common for clients who lived alone (82%) to use formal home help services, than for persons who lived with someone (24%) (p<0.001). |
| Larsson, 2006, Sweden | Cohort study (data collected from self and proxy report) | 652 people living alone (any type of dementia). Race/ethnicity NR. 87% female, 13% male. 100% living alone (in subgroup included in this review). Mean age reported for two groups: 86.2 and 86.94 (sd NR, range 81-100) | Stockholm, people living in ordinary dwellings (sample study as above) | Within subgroup living alone, compared those with dementia *vs.* without | Among people living alone, dementia doubled the likelihood of receiving home help (RR 2.03, p=0.043). |
| Larsson, 2004,  Sweden | Population-based sample, cross-sectional survey (data collected from self and proxy report, as well as medical records) | 520 participants (of those assigned to the dementia group, the diagnoses were AD, vascular dementia, and mixed or unspecified dementia). Race/ethnicity NR. 78% female, 22% male. 75% living alone. Mean age 86.70 (sd 3.65). | Stockholm; excluding residential care facilities or service apartments | Within subgroup living alone, compared with dementia *vs.* without | Fully adjusted multiple regression model: people living alone with dementia had higher odds of receiving home help (odds ratio = 4.60, 95% CI 1.43-14.86, p=0.011) compared to those living alone without dementia. |
| Schneider, 2002, UK | Cross-sectional (baseline of longitudinal study; data collected from self and proxy report) | 132 PWD (moderate-to-severe dementia). 93% white, others NR. 64% female. Mean age 81 (sd 7.7). | Secondary care caseloads (i.e. specialist dementia services) in South London | All had dementia, compared those living alone *vs.* with caregiver | People living with caregiver received more services overall than those living alone (mean 7.8 vs. 6.4, p=0.01). People living alone more likely to receive home help and meals on wheels. They received marginally more social care overall (p=0.057). No difference in respite care, day care, social worker. |
| Ebly, 1999,  Canada | Cohort study with follow-up at two years (data collected from self and proxy report) | 317 PWD (any dementia diagnosis). Race/ethnicity NR, 31.5% living alone. Live alone: 77% female, mean age 82.9 (sd 5.5). Not alone: 53.9% female, mean age 81.7 (sd 6.2). | National study of PWD whose caregivers agreed to be interviewed | All had dementia. Compared living alone *vs.* living with caregiver | No significant difference between groups in formal caregiving time received (living alone 22.3 hours per month, sd 44.6; not alone 19.9 hours per month, sd 48.4; p=0.68). Living alone more likely to receive meals service (24% vs. 4.2%, p<0.0001) and homemaker service (57% vs. 32.7%, p<0.0001). |
| Tuokko, 1999, Canada | Cross-sectional (two studies reported in single paper. Study 1: data collected from self report. Study 2: data collected from medical records) | Study 1: 177 people (possible or probable AD). Race/ethnicity NR (“most were Caucasian”). Gender NR. 40% living alone. Mean age, living alone group = 83.20. Mean age, if living with others and still married = 80.34. Mean age, widowed and living with others = 84.84. | Canadian Study of Health and Aging datasets – national prevalence study of dementia | All had dementia, compared living alone and not. | Those living alone used homemaker (p=0.001) and home-delivered meals (p=0.000) more frequently than those living with others. Use of other services did not differ significantly, but overall more people living alone used at least one service. Logistic regression: Living alone was significant predictor for using homemaker services (p=0.001), home-delivered meals (p<0.0001). Living alone increased odds of using home-delivered meals by 20 times. |
|  |  | Study 2: 152 PWD (any dementia). Race/ethnicity NR. 54% living alone. Living alone group: 76.8% female. Mean age 80.85 (sd 6.84). Living with spouse group: Gender NR. Mean age 75.01 (sd 6.10). | Two groups of clients selected from community psychogeriatric team | All had dementia, compared living alone *vs.* living with spouse. | People living alone used home-delivered meals more often than those living with spouses (p=0.021). No other differences in service use. Logistic regressions: no significant associations between service use and living arrangement. |
| Webber, 1994,  US | Cross-sectional (data collected from proxy report and medical records) | 2505 treatment center clients (diagnosis of possible or probable AD). 19.1% living alone. Living alone: 81% white, 1.3% Asian, 7.5% Black, 8.4% Hispanic, 84.1% female, 18.4% under 65, 22.6% age 65-74, 50.2% 75-84, 8.8% 85 and over. Living with others: 78.4% white, 2.8% Asian, 8.4% Black, 8.4% Hispanic, 63.6% female, 20.7% under 65, 29.3% 65-74, 41.3% 75-84, 8.7% 85 and over. | University-based Alzheimer’s Disease Diagnostic and Treatment Centers in California | All had AD, compared living alone *vs.* living with others. | More people living alone used home delivered meals (p<0.0005), homemaker chore service (p<0.0005), case management (p<0.0005). No difference in senior center (p values NR). Multivariate models: living alone was most consistently significant predictor of service use – meals (OR 2.01, CI 1.46-2.78), homemaker (OR 1.44, CI 1.10-1.89), case management (OR 2.74, CI 1.86-4.03). People living alone also more likely to use no services (p=0.14). |

**SPIDER TABLE OF INCLUSION AND EXCLUSION CRITERIA**

|  | **Inclusion criteria** | **Exclusion criteria** |
| --- | --- | --- |
| **Sample** | - Community-dwelling adults with cognitive impairment who live alone - Living alone defined as living in a non-institutional setting without others - Any diagnostic criteria accepted for dementia or cognitive impairment - No age limit imposed | - Studies conducted in assisted living facilities, nursing homes, and continuing care retirement communities - Studies that only measure subjective cognitive decline or subclinical memory problems associated with typical aging - Studies with a primary focus on other conditions associated with cognitive decline such as traumatic brain injury, stroke, Parkinson’s disease, and schizophrenia, due to their distinct patterns of functional and psychological complications |
| **Phenomenon of Interest** | - Use of health services, including but not limited to primary healthcare, inpatient services, outpatient services, emergency room services, dementia-specific/memory clinic - Use of social services, including but not limited to home health, adult day care, senior center, meal preparation/home-delivered meals/congregate meals, transportation, counseling, support groups, respite care, physical therapy, personal care/home help, homemaker services, information services, case management, outreach | - Studies not reporting on service use |
| **Design** | - Quantitative or mixed-methods studies reporting primary quantitative data | - Studies using qualitative methods only |
| **Evaluation** | - Studies that quantitatively measure use of services by people living alone with cognitive impairment | - Studies using qualitative methods only |
| **Research type** | - Peer-reviewed journal articles and grey literature - Full text available in English | - Review articles, opinion pieces, theoretical work, dissertations |

**QUALITY ASSESSMENT TABLES**

Quality assessment: Cross-sectional studies

| **First author, date** | **1.Were the criteria for inclusion in the sample clearly defined?** | **2.Were the study subjects and the setting described in detail?** | **3.Was the exposure measured in a valid and reliable way?** | **4. Were objective, standard criteria used for measurement of the condition?** | **5.Were confounding factors identified?** | **6.Were strategies to deal with confounding factors stated?** | **7. Were the outcomes measured in a valid and reliable way?** | **8. Was appropriate statistical analysis used?** | **Overall appraisal** |
| --- | --- | --- | --- | --- | --- | --- | --- | --- | --- |
| Henderson 2019 | **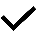** | **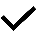** | **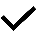** | **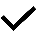** | **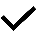** | **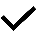** | **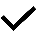** | **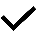** | Include |
| Moholt 2018 | **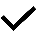** | **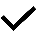** | **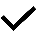** | ? | **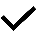** | **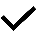** | **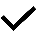** | **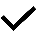** | Include |
| Rahja 2018 | **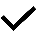** | **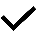** | **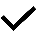** | **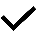** | **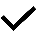** | **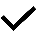** | **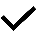** | **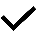** | Include |
| Michalowsky 2018 | **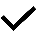** | **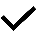** | **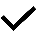** | **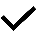** | **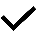** | **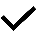** | ? | **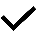** | Include |
| Gibson 2017 | **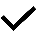** | **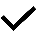** | **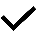** | **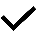** | **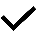** | **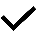** | ? | **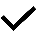** | Include |
| O’Brien 2017 | **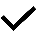** | **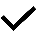** | **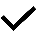** | **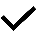** | **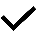** | **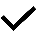** | ? | **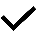** | Include |
| Eichler 2016 | **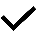** | **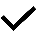** | **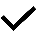** | **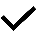** | **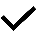** | **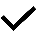** | ? | **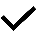** | Include |
| Michalowsky 2016 | **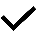** | **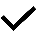** | **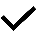** | **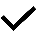** | **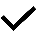** | **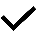** | ? | **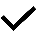** | Include |
| Alzheimer's Association  2012 | **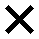** | **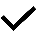** | ? | ? | ? | ? | ? | ? | Grey literature – include, with caveats |
| Takechi 2012 | **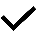** | **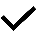** | **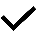** | **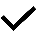** | **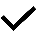** | **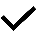** | **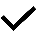** | **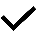** | Include |
| Miranda-Castillo 2010 | **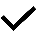** | **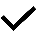** | **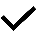** | **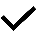** | **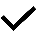** | **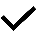** | **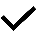** | **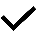** | Include |
| Edwards 2007 | **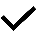** | **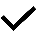** | **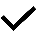** | **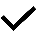** | **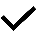** | **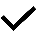** | **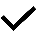** | **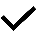** | Include |
| Larsson 2004 | **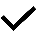** | **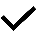** | **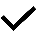** | **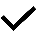** | **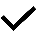** | **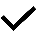** | ? | **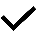** | Include |
| Schneider 2002 | **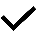** | **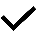** | **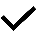** | **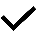** | **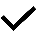** | **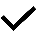** | **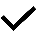** | **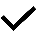** | Include |
| Ebly 1999 | **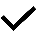** |  |  |  |  |  | ? |  | Include |
| Tuokko 1999 (study 1) |  |  |  |  |  |  | ? |  | Include |
| Tuokko 1999 (study 2) |  |  |  |  |  |  | ? |  | Include |
| Webber 1994 |  |  |  |  |  |  | ? |  | Include |
| Bass 1992 |  |  |  |  |  |  | ? |  | Include |

**QUALITY ASSESSMENT: COHORT STUDIES**

| **First author, date** | **1.Were the two groups similar and recruited from same population?** | **2.Were the exposures measured similarly to assign people to both exposed and unexposed groups?** | **3. Was the exposure measured in a valid and reliable way?** | **4. Were confounding factors identified?** | **5. Were strategies to deal with confounding factors stated?** | **6. Were the groups/ participants free of the outcome at the start of the study (or at the moment of exposure)?** | **7. Were the outcomes measured in a valid and reliable way?** | **8. Was the follow up time reported and sufficient to be long enough for outcomes to occur?** | **9. Was follow up completed, and if not, were the reasons to loss to follow up described and explored?** | **10. Were strategies to address incomplete follow up described and explored?** | **11. Was appropriate statistical analysis used?** | **Overall appraisal** |
| --- | --- | --- | --- | --- | --- | --- | --- | --- | --- | --- | --- | --- |
| Kerpershoe 2019 |  |  |  |  |  |  |  |  |  | N/A |  | Include |
| Huei-Ru 2017 |  |  | ? |  |  | N/A | ? |  | N/A | N/A |  | Include |
| Gage 2015 |  |  |  |  |  | N/A |  |  |  |  |  | Include |
| Soto 2015 |  |  |  |  |  | N/A | ? |  |  |  |  | Include |
| Wattmo 2014 |  |  |  |  |  | N/A | ? |  |  | ? |  | Include |
| Larsson 2006 |  |  |  |  |  | N/A |  |  |  |  |  | Include |
| Nourhashemi 2005 |  |  |  |  |  | N/A |  |  |  |  |  | Include |
| Schneider 2003 |  |  |  |  |  | N/A |  |  |  |  |  | Include |
| Chi 1995 |  |  |  |  |  |  |  |  |  | N/A |  | Include |

**QUALITY ASSESSMENT: CASE SERIES STUDIES**

| **First author, date** | **1.Were there clear criteria for inclusion in the case series?** | **2.Was the condition measured in a standard, reliable way for all participants included in the case series?** | **3.Were valid methods used for identification of the condition for all participants included in the case series?** | **4.Did the case series have consecutive inclusion of participants?** | **5.Did the case series have complete inclusion of participants?** | **6.Was there clear reporting of the demographics of the participants in the study?** | **7.Was there clear reporting of clinical information of the participants?** | **8.Were the outcomes or follow up results of cases clearly reported?** | **9.Was there clear reporting of the presenting site(s)/ clinic(s) demographic information?** | **10.Was statistical analysis appropriate?** | **Overall appraisal** |
| --- | --- | --- | --- | --- | --- | --- | --- | --- | --- | --- | --- |
| Knapp 2016 |  |  |  |  |  |  |  |  |  |  | Include |
| Thiruchselvam 2012 |  |  |  | ? | ? |  |  |  |  |  | Include |
| Wattmo 2011 |  |  |  | ? | ? |  |  |  |  |  | Include |
| Mavall 2007 |  |  |  |  | ? |  |  |  |  |  | Include |

**SAMPLE SEARCH STRATEGY**

Pubmed, 01/18/2020

((((("Cognition Disorders"[Mesh] OR "Dementia"[Mesh])) OR ((“cognitive impairment”[Title/Abstract] OR “cognitively impaired”[Title/Abstract] OR “cognitive disorder”[Title/Abstract] OR “cognitive decline”[Title/Abstract] OR “cognitive dysfunction”[Title/Abstract] OR “cognitive defect”[Title/Abstract] OR “memory loss”[Title/Abstract] OR “memory problem”[Title/Abstract] OR “memory problems”[Title/Abstract] OR “memory disorder”[Title/Abstract] OR “memory disorders”[Title/Abstract] OR “forgetful”[Title/Abstract] OR “confused”[Title/Abstract] OR “confusion”[Title/Abstract] OR dement*[Title/Abstract] OR alzheimer*[Title/Abstract] OR “primary progressive aphasia”[Title/Abstract] OR CADASIL[Title/Abstract] OR CARASIL[Title/Abstract] OR “Creutzfeldt Jakob”[Title/Abstract] OR “Frontotemporal lobar degeneration”[Title/Abstract] OR Huntington*[Title/Abstract] OR “Kluver Bucy”[Title/Abstract] OR “Picks disease”[Title/Abstract] OR “Pick’s disease”[Title/Abstract] OR “Primary Progressive Nonfluent aphasia”[Title/Abstract] OR “sundown syndrome”[Title/Abstract] OR sundowning[Title/Abstract] OR Korsakoff*[Title/Abstract] OR Binswanger*[Title/Abstract] OR “HIV-associated neurocognitive disorder”[Title/Abstract] OR “HIV associated neurocognitive disorder”[Title/Abstract] OR CJD[Title/Abstract] OR “AIDS Dementia Complex”[Title/Abstract] OR “Lewy body”[Title/Abstract] OR delirium[Title/Abstract] OR amnestic[Title/Abstract] OR “organic brain disease”[Title/Abstract] OR “organic brain syndrome”[Title/Abstract] OR “normal pressure hydrocephalus”[Title/Abstract] OR shunt[Title/Abstract] OR “benign senescent forgetfulness”[Title/Abstract]))))

AND

((((“living alone”[Title/Abstract] OR “live alone”[Title/Abstract] OR “lives alone”[Title/Abstract] OR “lived alone”[Title/Abstract] OR “home alone”[Title/Abstract] OR “living independently”[Title/Abstract] OR “live independently”[Title/Abstract] OR “lives independently”[Title/Abstract] OR “lived independently”[Title/Abstract] OR “living situation”[Title/Abstract] OR “living arrangements”[Title/Abstract]

OR “living arrangement”[Title/Abstract]))) OR "Independent Living"[Mesh]))

Filters: Publication date from 2018/08/01 to 2020/01/18

**PRISMA CHECKLIST**

| **Section/topic** | **#** | **Checklist item** | **Reported on page #** |
| --- | --- | --- | --- |
| **TITLE** | | |  |
| Title | 1 | Identify the report as a systematic review, meta-analysis, or both. | 1 |
| **ABSTRACT** | | |  |
| Structured summary | 2 | Provide a structured summary including, as applicable: background; objectives; data sources; study eligibility criteria, participants, and interventions; study appraisal and synthesis methods; results; limitations; conclusions and implications of key findings; systematic review registration number. | 4 |
| **INTRODUCTION** | | |  |
| Rationale | 3 | Describe the rationale for the review in the context of what is already known. | 6 |
| Objectives | 4 | Provide an explicit statement of questions being addressed with reference to participants, interventions, comparisons, outcomes, and study design (PICOS). | 6 |
| **METHODS** | | |  |
| Protocol and registration | 5 | Indicate if a review protocol exists, if and where it can be accessed (e.g., Web address), and, if available, provide registration information including registration number. | 7 |
| Eligibility criteria | 6 | Specify study characteristics (e.g., PICOS, length of follow-up) and report characteristics (e.g., years considered, language, publication status) used as criteria for eligibility, giving rationale. | 8-9 |
| Information sources | 7 | Describe all information sources (e.g., databases with dates of coverage, contact with study authors to identify additional studies) in the search and date last searched. | 7-8 |
| Search | 8 | Present full electronic search strategy for at least one database, including any limits used, such that it could be repeated. | 56 |
| Study selection | 9 | State the process for selecting studies (i.e., screening, eligibility, included in systematic review, and, if applicable, included in the meta-analysis). | 9 |
| Data collection process | 10 | Describe method of data extraction from reports (e.g., piloted forms, independently, in duplicate) and any processes for obtaining and confirming data from investigators. | 9 |
| Data items | 11 | List and define all variables for which data were sought (e.g., PICOS, funding sources) and any assumptions and simplifications made. | 9 |
| Risk of bias in individual studies | 12 | Describe methods used for assessing risk of bias of individual studies (including specification of whether this was done at the study or outcome level), and how this information is to be used in any data synthesis. | 10 |
| Summary measures | 13 | State the principal summary measures (e.g., risk ratio, difference in means). | N/A |
| Synthesis of results | 14 | Describe the methods of handling data and combining results of studies, if done, including measures of consistency (e.g., I^2^) for each meta-analysis. | N/A |

| **Section/topic** | **#** | **Checklist item** | **Reported on page #** |
| --- | --- | --- | --- |
| Risk of bias across studies | 15 | Specify any assessment of risk of bias that may affect the cumulative evidence (e.g., publication bias, selective reporting within studies). | N/A |
| Additional analyses | 16 | Describe methods of additional analyses (e.g., sensitivity or subgroup analyses, meta-regression), if done, indicating which were pre-specified. | N/A |
| **RESULTS** | | |  |
| Study selection | 17 | Give numbers of studies screened, assessed for eligibility, and included in the review, with reasons for exclusions at each stage, ideally with a flow diagram. | 10 and 43 |
| Study characteristics | 18 | For each study, present characteristics for which data were extracted (e.g., study size, PICOS, follow-up period) and provide the citations. | 11-12 |
| Risk of bias within studies | 19 | Present data on risk of bias of each study and, if available, any outcome level assessment (see item 12). | 11 |
| Results of individual studies | 20 | For all outcomes considered (benefits or harms), present, for each study: (a) simple summary data for each intervention group (b) effect estimates and confidence intervals, ideally with a forest plot. | 12-25 |
| Synthesis of results | 21 | Present results of each meta-analysis done, including confidence intervals and measures of consistency. | N/A |
| Risk of bias across studies | 22 | Present results of any assessment of risk of bias across studies (see Item 15). | N/A |
| Additional analysis | 23 | Give results of additional analyses, if done (e.g., sensitivity or subgroup analyses, meta-regression [see Item 16]). | N/A |
| **DISCUSSION** | | |  |
| Summary of evidence | 24 | Summarize the main findings including the strength of evidence for each main outcome; consider their relevance to key groups (e.g., healthcare providers, users, and policy makers). | 25-28 |
| Limitations | 25 | Discuss limitations at study and outcome level (e.g., risk of bias), and at review-level (e.g., incomplete retrieval of identified research, reporting bias). | 28-29 |
| Conclusions | 26 | Provide a general interpretation of the results in the context of other evidence, and implications for future research. | 29-30 |
| **FUNDING** | | |  |
| Funding | 27 | Describe sources of funding for the systematic review and other support (e.g., supply of data); role of funders for the systematic review. | 2 |

*From:*  Moher D, Liberati A, Tetzlaff J, Altman DG, The PRISMA Group (2009). Preferred Reporting Items for Systematic Reviews and Meta-Analyses: The PRISMA Statement. PLoS Med 6(7): e1000097. doi:10.1371/journal.pmed1000097

1. People with dementia [↑](#footnote-ref-1)
2. Not reported [↑](#footnote-ref-2)
3. Cognitive impairment [↑](#footnote-ref-3)
4. Alzheimer’s disease [↑](#footnote-ref-4)
5. Also reported on two other subgroups [↑](#footnote-ref-5)
6. Other races not reported [↑](#footnote-ref-6)
7. People with dementia [↑](#footnote-ref-7)
8. Not reported [↑](#footnote-ref-8)
9. Demographics also reported separately for subgroups with and without dementia [↑](#footnote-ref-9)
10. Also reported on two other subgroups [↑](#footnote-ref-10)
